# The Osteoimmunologic Basis of Biologic and Bioengineered Therapies in Osteoarthritis

**DOI:** 10.3390/biomedicines14081697

**Published:** 2026-07-28

**Authors:** Sarah Bergren, Hannah Shelby, Julian Wier, Edward M. Schwarz, Denis Evseenko, Jay R. Lieberman

**Affiliations:** 1 Department of Orthopaedic Surgery, Keck School of Medicine, University of Southern California, Los Angeles, CA 90033, USA; 2 Department of Orthopedics, Center for Musculoskeletal Research, University of Rochester Medical Center, Rochester, NY 14609, USA; 3 Alfred E. Mann Department of Biomedical Engineering, Viterbi School of Engineering, University of Southern California, Los Angeles, CA 90089, USA

**Keywords:** osteoarthritis, cartilage degeneration, osteoimmunology, scaffold, gene therapy, biomaterials, hydrogels

## Abstract

Osteoarthritis (OA) is a significant clinical problem that places a substantial burden on both patients and the healthcare system. Characterized by progressive cartilage degeneration, synovial inflammation, and subchondral bone remodeling, OA is a rapidly growing and increasingly studied disease affecting millions around the world. Growing evidence has expanded on the traditional view of OA as a mechanical “wear-and-tear” disease, highlighting that disease progression is driven not only by mechanical stress but also by chronic dysregulation of the osteoimmune environment. Activation of innate and adaptive immune pathways, macrophage M1 polarization, and dysregulated cytokine signaling all contribute to progressive joint degeneration. While current therapeutics often focus on managing symptoms or restoring joint mechanics, interventions often overlook the role of osteoimmunology in disease progression. This review summarizes the biological and bioengineering strategies emerging to address OA. These platforms include bioceramics, metal-based scaffolds, hydrogels, nanoparticles, and microsphere systems. Furthermore, small molecules, cell-based therapies, and gene-modified systems have also been shown to modulate the inflammatory microenvironment, enhance regulatory immune responses, and restore cartilage homeostasis. Together, these approaches represent the evolving research landscape, shifting away from just symptom alleviation and towards targeted disease-modifying therapies, including osteoimmunomodulation. However, significant barriers to clinical translation remain, such as limited large animal studies and species immune system differences, which need to be addressed for the development of clinically applicable interventions.

## 1. Introduction

Osteoimmunology is an integrative field that examines the dynamic interplay between the immune and skeletal systems [1]. Shared cytokines, immune cells, and signaling pathways regulate the delicate balance of bone remodeling and resorption [2]. Dysregulation of these immune–bone interactions contributes to alterations in bone structure and joint homeostasis, playing a central role in the pathogenesis of musculoskeletal pathologies such as osteoarthritis (OA) [3]. 

**Figure 1:**
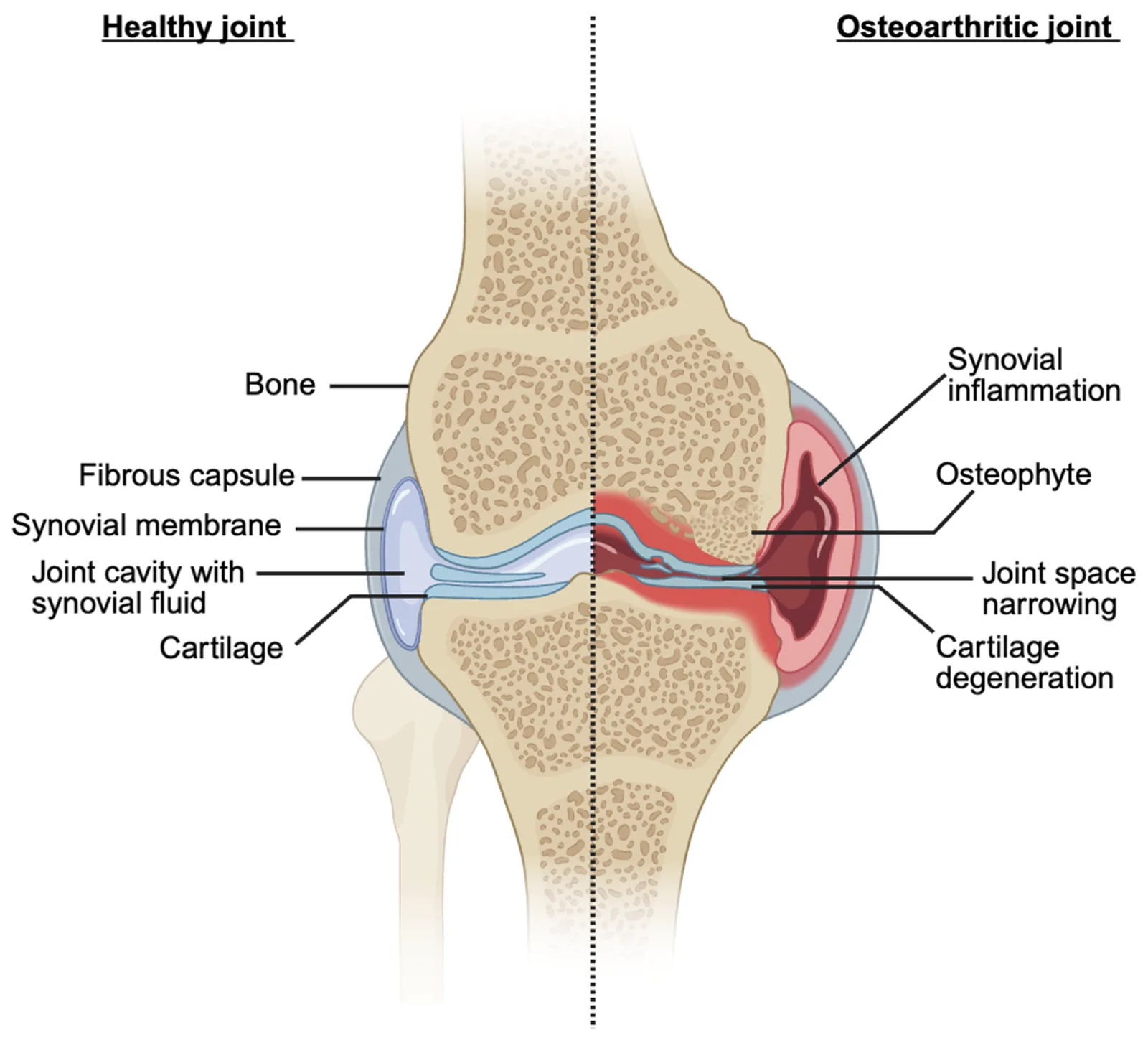
**Characteristics of OA.** The progression of OA is characterized by distinct radiographic features, including (1) joint space narrowing, (2) osteophyte formation, (3) subchondral cysts, and (4) subchondral sclerosis. Joint space narrowing occurs due to the degeneration of cartilage which eventually leads to direct contact between the tibia and femur. Osteophytes form due to the reactive activation of osteoblasts at the joint surface. Conversely, subchondral cysts and sclerosis develop as a result of increased mechanical forces on bone following the loss of cartilage, leading to microfractures and localized bone damage. Created in BioRender. Bergren, S. (2026) https://BioRender.com/qtmsdd3.

OA is a chronic degenerative joint disease characterized by progressive degeneration of articular cartilage, synovial inflammation, alterations in synovial fluid composition, and remodeling of subchondral bone (Figure 1). While traditionally viewed as a primarily mechanical “wear-and-tear” pathology, OA is now increasingly recognized as a complex “whole-joint” disease with substantial inflammatory and immunological components [4]. Emerging evidence suggests that activation of the immune system is a central driver of both OA initiation and progression, influencing cartilage breakdown, osteophyte formation, and subchondral bone sclerosis [5,6,7].

Both innate and adaptive immune responses contribute to OA pathophysiology [4]. Innate immune cells, including macrophages, mast cells, and neutrophils, respond to damage-associated molecular patterns (DAMPs) released from degraded cartilage and extracellular matrix, thereby triggering inflammatory signaling cascades within the synovium and subchondral bone [3]. Adaptive immune cells such as T and B lymphocytes also infiltrate the OA joint and shape the inflammatory milieu through cytokine secretion and direct cell–cell interactions [3]. This interplay perpetuates chronic inflammation, disrupting normal tissue repair and accelerating joint degeneration. Understanding how the immune system drives pathological bone remodeling and cartilage degradation provides critical mechanistic insight into disease progression and identifies opportunities for disease-modifying interventions that restore immune–skeletal homeostasis, rather than solely alleviating symptoms. As the field of osteoimmunology is still developing, this narrative review serves as a synthesis of the current research on osteoimmunologic therapies for OA to help better understand the direction and limitations of current potential therapeutic solutions. Important compounds, cell types, genes, proteins, scoring systems, molecules and pathways are defined in Table A1.

### 1.1. Clinical Relevance

OA is a leading cause of disability worldwide, particularly in aging populations. Symptomatic OA affected approximately 8% of people worldwide in 2020, and the absolute prevalence of OA has more than doubled over the past three decades, largely driven by the aging global population [8]. Further, while developing countries experience a decreased incidence of osteoarthritis compared to developed nations, the incidence of osteoarthritis is rapidly increasing worldwide, with a 9.2% increase from 1990 to 2021 [9]. 

OA imposes significant burdens on individuals and the healthcare system. Globally, OA is responsible for 2.2% of years lived with disability (YLDs), which equates to approximately 19 million years lived worldwide [10]. Over half of patients with OA report difficulty performing daily activities, such as household tasks, and fewer than 10% engage in the recommended weekly physical activity [11,12]. 

These functional limitations translate into considerable direct and indirect economic consequences. Patients with OA frequently experience work absenteeism and reduced productivity, with up to 20% of work time lost and an additional 30% reduction in on-the-job productivity [13,14]. From a societal perspective, the direct costs of OA are estimated to account for 1–2.5% of gross national product in developed countries, including the United States and Australia [15]. Collectively, the substantial societal burden and rising healthcare expenditures associated with OA underscore the urgent need to identify more effective and durable therapeutic strategies that address the underlying immune-driven mechanisms rather than solely focus on symptom control.

### 1.2. Therapeutic Gaps 

Clinical and translational data demonstrate that OA involves both mechanical wear and inflammation, whereby specific osteoimmunologic phenotypes drive both progression and pain. Single-cell and spatial studies reveal that synovial tissue from painful regions within OA joints harbors distinct fibroblast subsets that promote fibrosis, inflammation, and neurite outgrowth, thus directly linking stromal phenotypes to nociceptive remodeling [16]. Parallel work in synovial fluid identifies distinct immune phenotypes that predict divergent clinical trajectories in knee OA, while genetic studies implicate specific B-cell and monocyte traits as causal modifiers of hip and knee OA risk [17,18]. Recent clinical evidence demonstrates that higher intra-articular tumor necrosis factor-α (TNF-α) levels predict radiographic OA progression after hip arthroscopy, whereas lower synovitis burden and favorable cytokine signatures are associated with better outcomes after unicompartmental arthroplasty [19,20]. These results collectively support the potential of targeted therapy to improve postoperative outcomes and reduce the need for further surgery [19,20]. As current clinical strategies are primarily focused on symptomatic pain control, there is a critical need for strategies that enhance both the efficacy and durability of therapeutic responses. Joint inflammation predicts central and peripheral pain sensitization, with the loss and exhaustion of regulatory macrophage subsets predicting greater pain [21,22]. At the neuroimmune interface, cartilage-derived DAMPs can directly activate nociceptors, driving OA hyperalgesia [23,24]. Presently, systemic anti-inflammatories, steroid injections, and biologics fail to provide consistent and complete symptomatic relief and are unable to address the underlying pathogenesis; thus, identifying and targeting immune profiles may enhance the efficacy of these interventions. To that end, Tonutti et al. demonstrated unique peripheral blood inflammatory phenotypes in responders and non-responders to platelet-rich plasma injections in the knee joint, suggesting a potential for a targeted approach [25]. Together, these data motivate bioengineering strategies designed to reprogram local and systemic inflammatory phenotypes, modulate disease-specific immune cell networks, and prevent structural progression rather than merely treating symptoms.

## 2. Chronic Inflammation in OA

**Figure 2:**
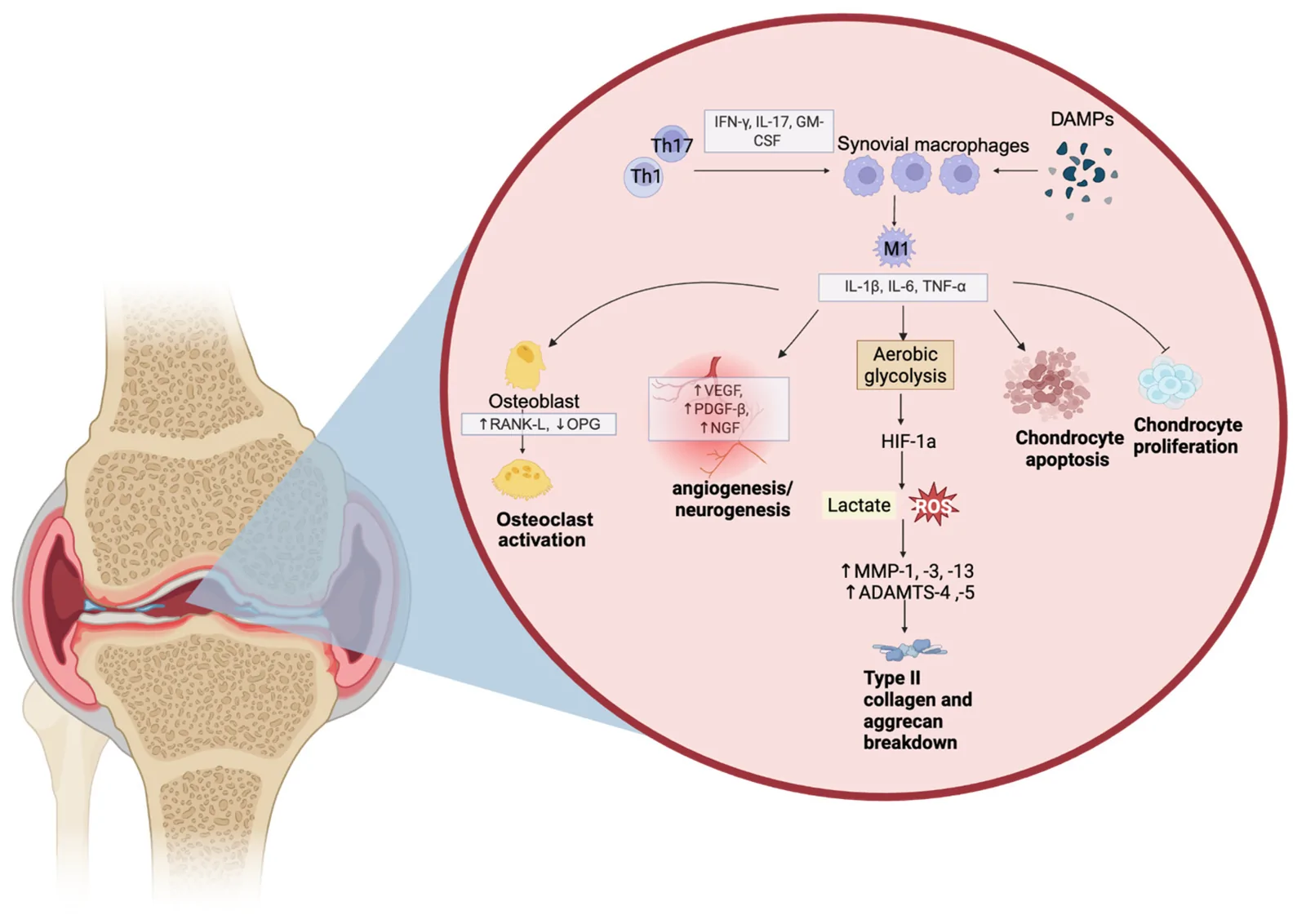
**Chronic inflammation in OA with cartilage degradation.** The chronic inflammatory environment characteristic of OA causes progressive damage to chondrocytes leading to the release of DAMPs (damage-associated molecular patterns) from surrounding cells, which polarize synovial macrophages toward the pro-inflammatory M1 phenotype [27]. M1 macrophages release inflammatory cytokines IL-1β, IL-6 and TNF-α which drive degradation of type II collagen and aggrecan within chondrocytes and synoviocytes through activation of matrix metalloproteinases (MMPs) and aggrecanases (ADAMTSs) [28]. Exposure to TNF-a and IL-1β also shift cells to aerobic glycolysis, which stabilizes hypoxia-inducible factor-1 alpha (HIF-1α). This amplifies the transcription of matrix-degrading enzymes, increases lactate and reactive oxygen species production, impairs matrix integrity and decreases chondrocyte viability [29,30]. Furthermore, IL-1β and TNF-α inhibit the proliferation of chondrocytes while inducing apoptosis [31]. Within the subchondral bone, inflammatory cytokines lead to increased expression of RANKL and suppress OPG, causing osteoclast activation and bone resorption [32]. As bone resorption occurs, the resulting mechanical instability leads to osteophyte formation and sclerosis. Additionally, angiogenic and neurogenic factors (VEGF, PDGF-β and NGF) perpetuate pain and vascularization within the subchondral bone [33]. The immune system amplifies this further through adaptive immune cell activation (Th1 and Th17 lymphocytes), which release inflammatory molecules (IFN-γ, IL-17, and GM-CSF) to maintain macrophage M1 polarization, cartilage degradation and osteoclastogenesis [3,34]. Created in BioRender. Bergren, S. (2026) https://BioRender.com/qtmsdd3.

The pathogenesis of OA is closely linked to the immune system, in which the activation of immune cells leads to progressive joint and cartilage degeneration [3] (Figure 2). In healthy joints, transient immune signaling contributes to tissue maintenance and repair; however, OA is associated with persistent low-grade inflammation that sustains a pathological interaction between synovial immune cells, chondrocytes, and subchondral bone [26].

Within the joint, macrophages serve as immune regulatory mediators, influencing the balance between pro-inflammatory and pro-healing cytokines. Pattern recognition of DAMPs released by damaged cartilage into the synovium polarizes macrophages toward a predominantly pro-inflammatory M1 phenotype, in which cytokines such as interleukin-1β (IL-1β), IL-6, and TNF-α activate nuclear factor kappa-light-chain-enhancer of activated B cells (NF-κB) signaling in chondrocytes and synoviocytes [3]. This cascade induces matrix metalloproteinase (MMP-1, MMP-3, MMP-13) and aggrecanase (ADAMTS-4 and -5) expression, accelerating the breakdown of type II collagen and aggrecan [26]. Simultaneously, IL-1β and TNF-α inhibit chondrogenic progenitor cell proliferation and promote chondrocyte apoptosis, limiting intrinsic repair capacity. Although M2 polarization by IL-4 and IL-13 promotes resolution, OA exhibits a sustained M1-M2 imbalance that perpetuates cytokine production and tissue injury [3,26]. Of note, while macrophage polarization is traditionally delineated as M1 (pro-inflammatory) or M2 (pro-reparative), this is not binary but rather a spectrum with macrophages showing a combination of inflammatory and anti-inflammatory properties (i.e., M2a, M2b) [35,36]. 

Cellular metabolism, specifically glycolysis, plays a key role in sustaining chronic inflammation in OA. In healthy tissue, macrophages and chondrocytes predominantly rely on oxidative phosphorylation; however, exposure to TNF-α and IL-1β induces a metabolic shift towards aerobic glycolysis [30]. This transition stabilizes hypoxia-inducible factor-1 alpha (HIF-1α), which in turn, amplifies transcription of IL-1β, IL-6, and matrix-degrading enzymes, worsening synovial and cartilage inflammation [29]. This glycolytic shift also increases lactate and reactive oxygen species production, which further accounts for impaired matrix integrity and decreased chondrocyte viability [37]. Notably, restoring oxidative metabolism has been reported to increase M2 polarization and limit pro-inflammatory cytokine release [29]. 

Beyond the cartilage surface, osteoimmunologic crosstalk extends to subchondral bone, where cytokines stimulate osteoblasts to express receptor activator of nuclear factor kappa-b ligand (RANKL) and suppress osteoprotegerin (OPG), shifting the balance toward osteoclastogenesis and subchondral bone resorption [38]. The resulting microarchitectural instability triggers compensatory sclerosis and osteophyte formation, further distorting joint biomechanics. Meanwhile, angiogenic and neurogenic mediators such as vascular endothelial growth factor (VEGF), platelet-derived growth factor-β (PDGF-β), and nerve growth factor (NGF) couple inflammation to aberrant vascular and sensory nerve ingrowth, perpetuating pain and cytokine release [33]. Adaptive immune responses compound this cycle further, whereby specific T helper cells, Th1 and Th17, infiltrate the synovium, secreting interferon-γ (IFN-γ), IL-17, and granulocyte-macrophage colony-stimulating factor (GM-CSF), which reinforces macrophage activation and matrix degradation [34]. In this highly inflammatory environment, regulatory T cells and anti-inflammatory T helper cells (Th2) are numerically and functionally diminished, further driving the microenvironment towards an inflammatory state [34]. Collectively, this failure of immunologic resolution transforms the joint into a chronic “smoldering” inflammatory niche in which innate and adaptive immune circuits drive continuous catabolic signaling in cartilage and subchondral bone [39]. These osteoimmune mechanisms explain the progression of OA from an episodic, injury or load-driven pathology to a self-perpetuating disease. Importantly, this framework also highlights the therapeutic potential of immune modulation, particularly in targeting macrophage polarization, cytokine networks, and RANKL signaling, as a promising avenue for disease-modifying therapies. 

## 3. Immunology and Cartilage Regeneration

There is a complex interplay between immune cells and chondrogenic cells that regulates cartilage repair and the progression of OA. While once thought to be immune-privileged due to its avascularity, cartilage homeostasis and the immune system are now known to be intertwined, sharing common pathways and molecules [40,41]. For example, pro-inflammatory cytokines, including IL-1β, TNF-α, and IL-6, are involved in the breakdown of cartilage extracellular matrix and the synovial inflammation characteristic of OA [42,43]. In contrast, IL-10 and transforming growth factor-β (TGF-β) are essential for chondrocyte development and proliferation, supporting reparative pathways by enhancing the synthesis of type II collagen and aggrecan [43,44]. 

**Figure 3:**
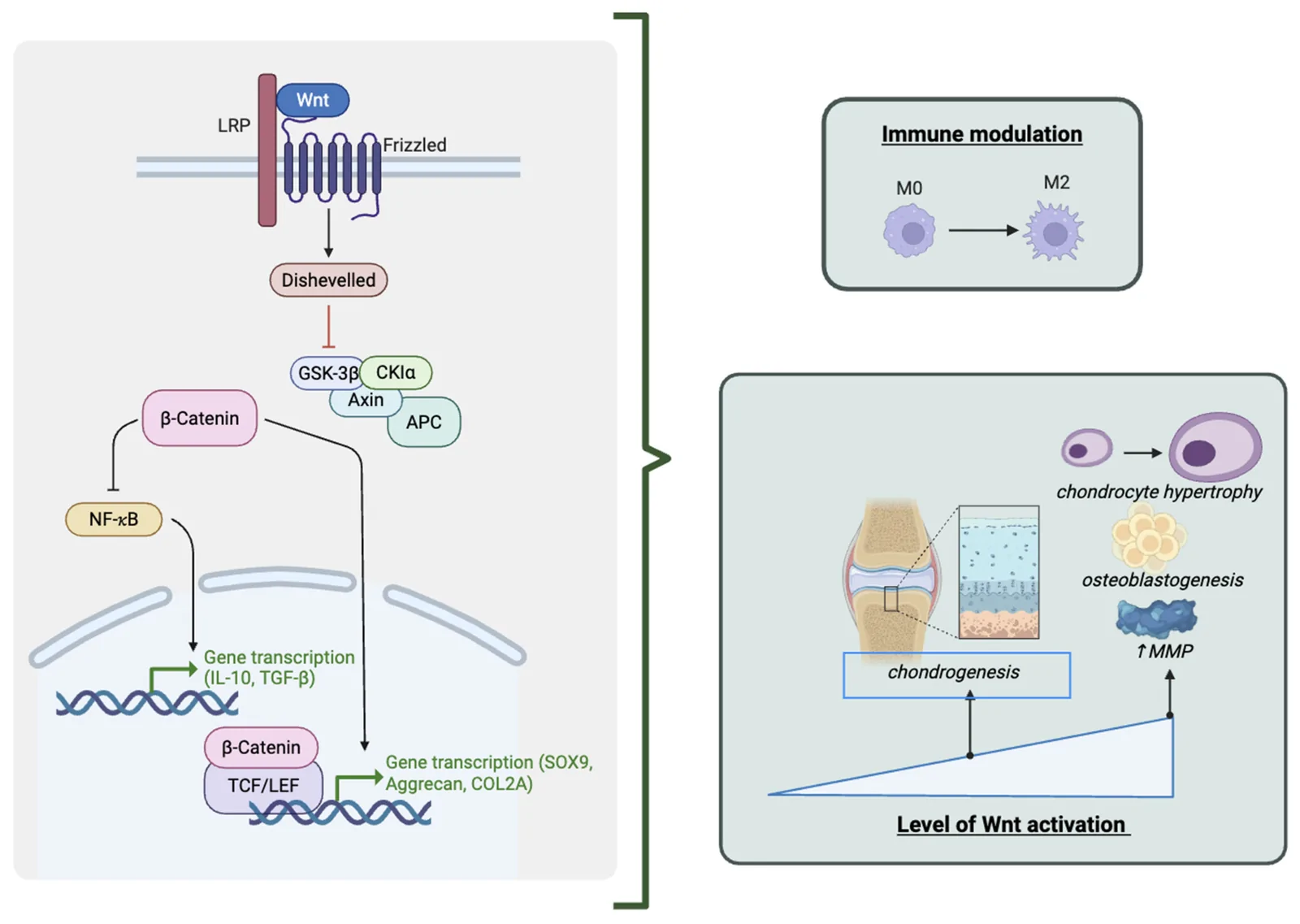
**The role of the Wnt pathway in immune regulation and chondrocyte homeostasis.** Activation in the Wnt pathway leads to suppression of NF-κB and increases transcription of essential chondrogenic genes. When the Wnt pathway is active, the disheveled protein inhibits the β-catenin destruction complex, resulting in the accumulation of β-catenin [51]. This accumulation of β-catenin inactivates NF-κB, decreasing the expression of pro-inflammatory cytokines (such as TNF-α and IL-1β) and increasing IL-10 and TGF-β levels, which promotes M2 macrophage polarization [45,46]. Additionally, β-catenin translocates into the nucleus and forms a complex with T-cell factor/lymphoid enhancer factor (TCF/LEF) transcription factors, driving the expression of SRY-box transcription factor 9 (SOX9), aggrecan (ACAN) and type II collagen (COL2A1) and promoting chondrogenesis [47]. However, overactivation of the Wnt pathway within cartilage can lead to joint degradation by inducing chondrocyte hypertrophy, stimulating osteoclastogenesis and activating MMPs [47]. Created in BioRender. Bergren, S. (2026) https://BioRender.com/qtmsdd3.

The Wnt/β-catenin pathway also plays a distinct role in immune system regulation and cartilage homeostasis, acting as a key mediator of both immune cell and chondrocyte phenotypes (Figure 3). Within the immune system, the canonical Wnt pathway can promote the pro-healing M2 phenotype by inhibiting NF-*κ*B signaling, thereby enhancing the production of anti-inflammatory cytokines such as IL-10 and TGF-β [45,46]. Within cartilage, a basal level of Wnt signaling is required for chondrogenesis; however, overactivation of this pathway leads to osteoblastogenesis, chondrocyte hypertrophy, and upregulation of MMPs, thereby causing cartilage matrix degradation [47]. The importance of maintaining this balance has been demonstrated experimentally. In a mouse model, continuous activation of β-catenin in chondrocytes was found to cause articular cartilage loss and osteophyte formation in joints [48]. Conversely, in another mouse model, continuous inhibition of β-catenin has also been associated with increased articular cartilage degeneration [49]. This is likely due to β-catenin’s effect on chondrocyte maturation: increased levels accelerate maturation, while decreased levels delay it [48]. Additionally, specific gene mutations have been reported to influence the development and progression of OA. Single-nucleotide polymorphisms in the sFRP3 gene, an antagonist of the Wnt pathway, have been discovered that increase the risk for OA, and patients with knee OA have been observed to have lower levels of Dickkopf-1, another inhibitor of this pathway [50].

**Figure 4:**
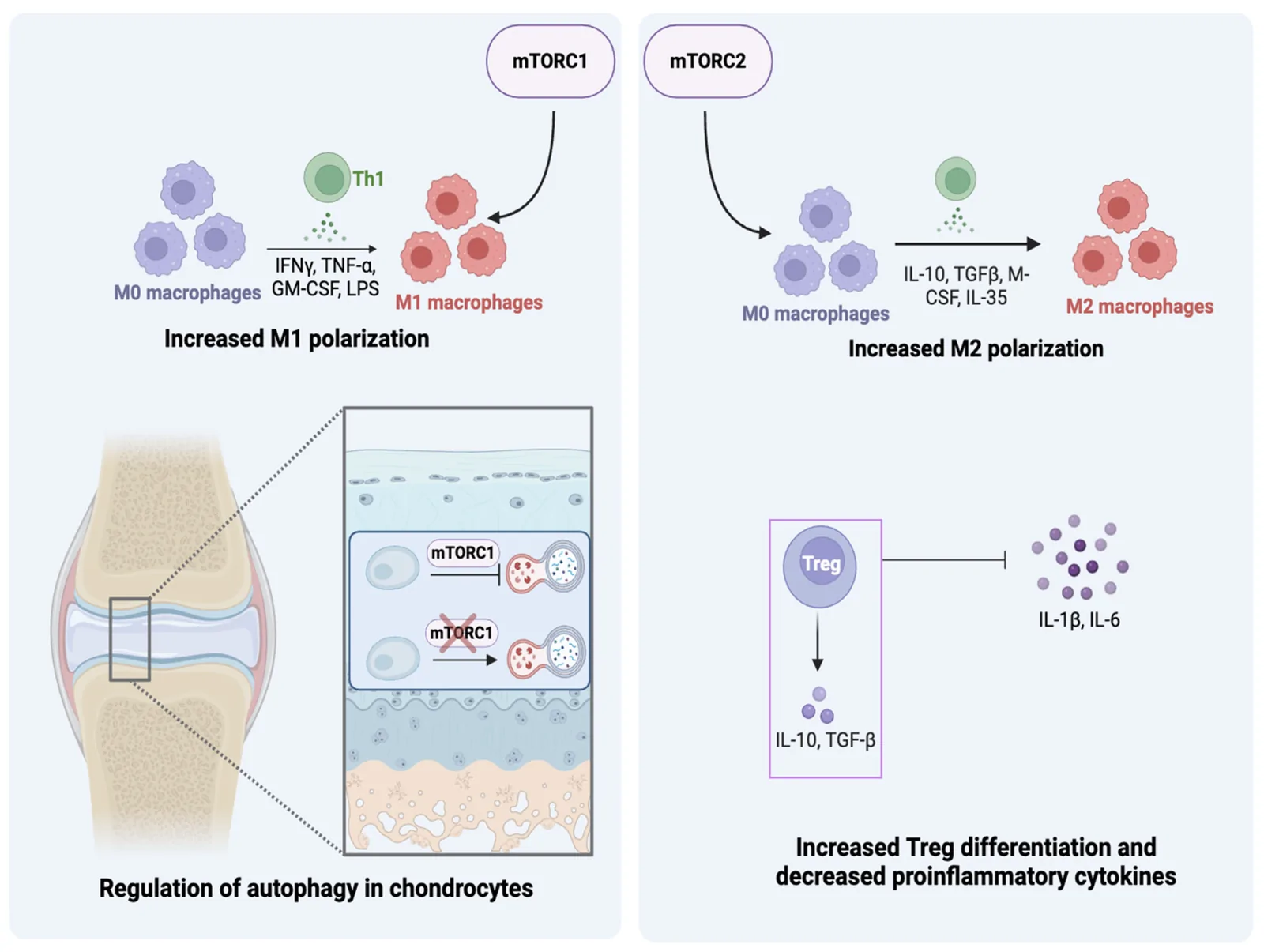
**The impact of mTORC1 vs. mTORC2 on macrophage polarization and chondrocyte autophagy.** The mammalian target of rapamycin (mTOR) pathway plays a key role in both immune system regulation and chondrocyte homeostasis. The mTOR pathway consists of two complexes, mTOR complex 1 (mTORC1) and mTORC2. mTORC1’s activity is context dependent and when chronically activated mTORC1 promotes inflammation and suppresses chondrocytes autophagic activity. Activation of mTORC1 during acute inflammation leads to increased M1 macrophage polarization through the upregulation of pro-inflammatory cytokines such as IFN-γ and TNF-α [52]. mTORC1 also inhibits chondrocyte autophagy, a cellular process that removes damaged proteins and organelles to maintain cellular function [56]. The inhibition of mTORC1 is associated with enhanced cartilage repair and reduces joint degeneration. Conversely, mTORC2 is frequently associated with creating a pro-healing environment by increasing M2 macrophage polarization and promoting the development of regulator T-cells, which inhibit inflammatory cytokines and enhance anti-inflammatory signaling [52,53]. Created in BioRender. Bergren, S. (2026) https://BioRender.com/qtmsdd3.

Likewise, the mammalian target of rapamycin (mTOR) pathway regulates the metabolism of immune cells and chondrocytes (Figure 4). Specifically, mTOR coordinates the metabolic regulation of T-lymphocytes and macrophages, functioning as a central regulator of cell activation, growth, and development [52,53,54,55]. The mTOR pathway is a complex, context-dependent pathway. Within the immune system, mTOR acts through two complexes: mTOR complex 1 (mTORC1) and mTOR complex 2 (mTORC2). mTORC1 can influence M1 or M2 macrophage polarization, depending on the microenvironment, nutrient availability and the cytokines present [52]. Under nutrient-rich conditions, mTORC1 can promote the polarization of macrophages towards the inflammatory M1 phenotype, particularly when in acute inflammatory environments, and activate effector T-cells [52]. mTORC2 is typically associated with an anti-inflammatory, reparative environment. mTORC2 can influence M2 macrophage polarization and the promotion of regulatory T-cell (Treg) differentiation [52,53]. By promoting Treg differentiation, the mTOR pathway can suppress T-cell activation and reduce inflammation locally [53]. In cartilage homeostasis, mTOR serves as the main inhibitor of cartilage autophagy. Autophagy is a cytoprotective process that improves chondrocyte survival by removing long-lived or damaged organelles and proteins [56]. As OA progresses, pathologic activation of the mTOR signaling cascade suppresses autophagy, leading to increased oxidative stress within chondrocytes, cellular senescence, and apoptosis [56]. 

Signaling pathways such as NF-κB, Wnt/β-catenin and mTOR are interconnected with shared cytokines and signaling molecules to modulate the development of osteoarthritis and macrophage polarization. Pro-inflammatory molecules such as TNF-α and IL-1β activate both NF-κB and mTORC1 to amplify inflammatory cytokine expression, promote M1 macrophage polarization and promote cartilage degradation [35,57]. Conversely, both mTORC2 and the conical Wnt/β-catenin pathway can promote M2 macrophage polarization through increased expression of IL-10 and TGF-β [45,52]. The Wnt and mTOR pathways are also directly connected through the disheveled proteins. Autophagy downregulates the Wnt pathway by reducing disheveled levels; however, mTORC1 can inhibit autophagy and therefore play a role in Wnt pathway activation [58]. However, given the context-dependent role of mTORC1, this complex has also been shown to inhibit the Wnt/β-catenin pathway through enhancing the internalization of the Frizzled receptor protein [59]. The relationship between these different pathways is complex and is still not yet fully understood. 

Given the central roles of the Wnt and mTOR pathways in immunomodulation and cartilage repair and degradation, therapeutic strategies targeting these pathways may confer dual benefits by reprogramming the local immune microenvironment while simultaneously preserving chondrocyte viability and anabolic capacity. Understanding the interaction between the immune system and cartilage homeostasis is important for the rational design of biologic scaffolds that promote regenerative signaling targeting osteoimmunologic mediators while also providing chondrogenic elements and mechanical support. 

## 4. Therapeutic Solutions

Biomaterial-based therapies, particularly bioceramics, metal-based scaffolds, hydrogels, nanoparticles, and microspheres, have emerged as promising strategies in the management of OA due to their ability to deliver drugs locally and in a responsive manner within the joint space (Table 1). Bioceramics and metal scaffolds play a key role due to their material stability and their capacity for controlled delivery of bioactive and immunomodulatory molecules. Hydrogels and nanoparticles are highly customizable therapeutics that can conform to the natural shape of articular surfaces and sustain the release of therapeutic agents in the OA environment, characterized by chronic, low-grade inflammation [60].

To test emerging therapies, it is necessary to use accurate, relevant animal models; however, developing OA models applicable to humans poses challenges. OA is a complex, progressive disease process with multiple causes and stages; thus, a unified animal model remains elusive. As such, different etiologies of OA initiation and progression have been modeled to capture the pathophysiological breadth of OA. Idiopathic, age-related degeneration can be induced by intra-articular injection of compounds such as monosodium iodoacetate, but these models are less frequently used in biomaterial-based research. Post-traumatic OA (PTOA) models are more commonly used (e.g., medial meniscus transection, anterior cruciate ligament transection), and they share the core inflammatory and immune mechanisms present in OA [61]. These models offer a clear initiation time point, allowing researchers to track the progression of the disease. Cartilage defect models represent a subset of PTOA and are frequently used to evaluate the efficacy of therapeutics designed to promote cartilage regeneration [62,63,64]. These models are also often used as examples of acute injury that can lead to early OA and are the most common models used in biomaterial-based research related to OA [64]. Additionally, osteochondral defects are not only representative of the disease process but are also a serious downstream consequence of the degenerative process and, therefore, an important consideration when developing a therapeutic treatment for OA [65]. Although these models fail to directly capture each subcomponent of OA pathogenesis, they involve key biological processes involved in cartilage repair and regeneration [66]. This review includes studies using all three model types. 

**Table 1:** Summary of recent therapeutic studies for osteoarthritis.

Study	Translational Stage	Population/Model	N	Intervention	Osteoimmunologic Target	Outcomes	Reference
Zhai et al., 2020	In vitro	Mouse macrophages and rabbit chondrocytes	–	Bioceramic (lithium-calcium silicate)	Macrophage polarization; Cytokine modulation	- Developed lithium–calcium silicate bioceramics, which released Li and Si ions in a controlled manner - Ion release downregulated pro-inflammatory cytokines (TNF-α, IL-6, IL-1β) and upregulated IL-10, inducing M2 macrophage polarization - Enhanced chondrocyte proliferation and matrix production (type II collagen, aggrecan) - Promoted osteochondral repair, increased glycosaminoglycan (GAG) content, and improved interface organization	[67]
Lin et al., 2019	Small animal	Rabbit femoral condyle osteochondral defect model	24	Bioceramic (copper- incorporated bioactive glass)	Macrophage polarization	- Manufactured copper-incorporated bioactive glass-ceramics (Cu-BGC) with sustained release of Cu^2+^ ions - Macrophages shifted to M2 anti-inflammatory phenotype - M2 macrophages secreted IL-10, enhancing chondrocyte matrix synthesis and MSC osteogenic differentiation - In a rabbit osteochondral defect model, Cu-BGC significantly improved cartilage thickness, type II collagen deposition, and subchondral bone regeneration while reducing synovial inflammation	[68]
Deng et al., 2022	Small animal	Rabbit knee osteochondral defect model	24	Bioceramic (cerium)	Reactive oxygen species modulation	- Engineered cerium-doped bioceramic scaffolds with intrinsic redox cycling (Ce^3+^/Ce^4+^) capacity - Scaffolds scavenged reactive oxygen species, reducing oxidative stress - Reactive oxygen species reduction decreased IL-1β/TNF-α and improved chondrocyte viability - In vivo, cerium-bioceramics improved cartilage preservation, increased type II collagen, and enhanced subchondral bone regeneration	[69]
Y. Chen et al., 2024	Small animal	Rabbit knee OA model (full-thickness cartilage defect)	12	Metal Scaffold (metal–phenolic network on a 3D scaffold)	Inflammatory cytokine modulation; Catabolic enzyme inhibition	- A epigallocatechin gallate and strontium-based metal phenolic network-modified 3D scaffold was implanted in the defect site of a rabbit OA model - In vitro, the scaffold decreased expression of MMP-13, IL-1β, TNF-α and ADAMTS5 and increased type II collagen and aggrecan levels after treatment with IL-1β - The scaffold leads to cartilage integration after 12 weeks, diminished chondrocyte apoptosis, increased extracellular matrix deposition and increased COL2A1 expression	[70]
Xu et al., 2025	Small animal	Rat knee OA model (medial meniscus destabilization)	20	Metal Scaffold (magnesium nanoparticles)	Macrophage polarization	- A rodent OA model was injected weekly with a hydrogel containing magnesium-based nanoparticles - Found to inhibit M1 macrophage polarization and promote M2 phenotype - Hydrogel-treated rats were observed to have improved joint spacing, preservation of cartilage and sustained COL2A1 expression	[71]
Khader & Arinzeh, 2020	In vitro	Human MSCs	-	Metal Scaffold (zinc oxide composite scaffold)	MSC differentiation	- Proof of concept of a zinc oxide composite scaffold for osteochondral healing - The differentiation of MSCs into chondrocytes was enhanced at lower zinc oxide concentrations with increased expression of SOX9 and COL2A1 - Osteogenesis was enhanced at higher zinc oxide concentrations with increased RUNX2 and SP7 (osterix) expression	[72]
Deng et al., 2025	Small animal	Mouse knee OA model (intra-articular injection of monosodium iodoacetate)	40	Metal Scaffold (Mn or Co ions within akermanite microparticles [AKT-MPs])	Macrophage polarization; Reactive oxygen species modulation; Catabolic enzyme inhibition	- In vitro, Mn or Co-AKT-MPs demonstrated the ability to clear reactive oxygen species, suppress M1 macrophage polarization, decrease cartilage ECM loss and reduce the secretion of degradation enzymes compared to controls - In vivo, mice injected with Mn or Co-AKT-MPs experienced decreased synovial inflammation and slowing of OA progression compared to untreated controls	[73]
Sang et al., 2022	Small animal	Rat knee OA model (meniscus resection)	50	Hydrogel (Pluronic F-127 and hyaluronic acid)	Macrophage polarization; Cartilage degeneration	- A thermosensitive hydrogel was developed through crosslinking Pluronic F-127 and hyaluronic acid - Utilized as a carrier for the sustained release of primary chondrocyte-derived exosomes - In a murine OA model, researchers observed higher COL2A1 and RUNX1 expression, an increase in the M2-to-M1 ratio, and decreased cartilage degeneration on histology	[74]
Lu et al., 2024	Small animal	Rat knee OA model (full-thickness cartilage defect at the distal femoral condyle)	33	Hydrogel (phenylboronicacid-crosslinked polyvinyl alcohol and hyaluronic acid)	Macrophage polarization; Reactive oxygen species modulation	- Upper-hydrogel involved phenylboronic-acid-crosslinked polyvinyl alcohol, which had the ability to degrade and elute diclofenac when exposed to reactive oxygen species, reducing oxidative stress - Lower hydrogel made of hyaluronic acid was able to release stem cell derived exosomes at the site of injury, resulting in chondrogenesis - Within a murine OA model, researchers observed a higher M2-to-M1 ratio, with uniform cartilage regeneration on histology	[75]
Jiang et al., 2023	Small animal	Rabbit knee OA model (full thickness cartilage defect)	8	Hydrogel (methacrylated gelatin and hyaluronic acid hydrogel with zeolitic imidazolate frameworking containing neobavaisoflavone)	Macrophage polarization; Cytokine modulation	- In a rabbit OA model, a methacrylated gelatin and hyaluronic acid hydrogel containing neobavaisoflavone encapsulated in zeolitic imidazolate frameworks was found to effectively regenerate cartilage similar to native tissue - The hydrogel allowed for effective delivery of neobavaisoflavone - Demonstrated decreased inflammatory cell infiltration, increased M2 polarization, and increased TGF-β3 expression, leading to an anti-inflammatory microenvironment that promoted regeneration.	[76]
S. Xu et al., 2022	Large animal model	Goat subcutaneous implantation model	6	Hydrogel (chitosan [CTS] hydrogel loaded with icariin [ICA])	Cytokine modulation	- Icariin, a plant-based compound, has been noted to have anti-inflammatory and chondrogenic properties - In vitro, the ICA/CTS hydrogel accelerated chondrocyte proliferation, enhanced chondrogenesis and decreased expression of inflammatory cytokines (IL-6 and TNF-α) compared to CTS hydrogels - In vivo, the ICA/CTS hydrogel demonstrated an increase in GAG and COL2A1 expression as well as a more typical lacuna-like structure compared to CTS hydrogel at both 3 and 6 weeks post-implantation.	[77]
Li et al., 2025	Small animal	Mouse knee OA model (destabilization of the medial meniscus)	40	Nanoparticle (IL-10/NO- prednisolone)	Cytokine modulation	- IL-10/NO-prednisolone nanoparticles targeted synovial macrophages, reducing glycolysis and cartilage loss - Interrupted glycolytic signaling cascade by modulating the IL-10, IL-10Rα, and the HIF-1α axis, which suppressed glycolysis, reduced reactive oxygen species and lactate accumulation, and limited chondrocyte ferroptosis - Results revealed evidence of improved proteoglycan content, improved Safranin-O staining and lower cartilage degeneration scores	[78]
Qiu et al., 2024	Small animal	Rat knee OA model (medial meniscectomy)	50	Microsphere (parecoxib/IL-4)	Cytokine modulation	- Double-layer microspheres released parecoxib (early) and IL-4 (sustained) - Found to reduce IL-1β/TNF-α, increase M2 macrophage proportion, and enhance cartilage matrix deposition and joint structure	[79]
Dai et al., 2025	Small animal	Rat knee OA model (ACL transection and medial meniscectomy)	30	Microsphere (aggregation- induced emission nanomicelles)	Macrophage polarization; Reactive oxygen species modulation	- Developed microfluidic microspheres incorporating AIE (aggregation-induced emission) nanomicelles - AIE nanomicelles acted as reactive oxygen species scavengers and anti-inflammatory agents, while also emitting fluorescence for cartilage imaging - Found to lower synovial inflammation, decrease pro-inflammatory macrophages, and reduce oxidative stress inside the joint - Cartilage outcomes included improved Safranin-O staining, enhanced type II collagen deposition, and reduced cartilage erosion compared to controls	[80]
Ko et al., 2013	Small animal	Rat knee OA model (ACL transection)	36	Microspheres (sulforaphane– PLGA)	Catabolic enzyme inhibition	- Sulforaphane (SFN) encapsulated in biodegradable PLGA microspheres - In vitro, SFN-PLGA reduced LPS-induced COX-2, ADAMTS-5, and MMP-2 gene and protein expression - In vivo, intra-articular SFN-PLGA delayed cartilage degeneration, preserved type II collagen, and reduced collagen type X expression and synovial inflammation with no systemic toxicity	[81]
Lohmander et al., 2014	Clinical	Patients with knee OA	192	Small molecule (rhFGF-18)	Catabolic enzyme inhibition	- Patients were administered a single or multi-dose regimen of 10 μg, 30 μg, or 100 μg of Sprifermin or a placebo - Intra-articular injection of Sprifermin lead to no significant differences in central medial femorotibial cartilage thickness between groups - There was a dose dependent effect on lateral femorotibial cartilage thickness change, with the multi-dose 100 μg Sprifermin group demonstrating a significant reduction in cartilage loss at 12 months compared to the placebo group - All patients in this study experienced improvements in WOMAC scores; however, patients receiving the placebo experienced greater improvements compared to patients receiving Sprifermin	[82]
Shkhyan et al., 2023	Small animal; Large animal	Multiple models: Mouse knee OA model (destabilization of medial meniscus); Rat knee OA model (medial meniscal tear model), Canine OA model (meniscal release)	20 -24	Small molecule (R805 [CX-011])	Inflammatory signaling pathway inhibition; Macrophage polarization; Fibroblast polarization	- In a mouse model, researchers demonstrated that inactivation of the non-canonical gp130 signaling mediated by the SRC family of kinases can decrease proteoglycan loss, synovitis and fibrosis compared to controls - Developed a small molecule R805, which can be injected intra-articularly to pharmacologically inactive gp130 and the associated signaling pathway - In rat and canine OA models, the injection of R805 resulted in decreased cartilage degradation and inflammation as shown histologically. In the canines specifically, there was also decreased pain and lameness scores compared to controls - Single-cell sequencing data from cells isolated from rat and dog models showed an increased proportion of macrophages and fibroblasts associated with an anti-inflammatory phenotype	[83]
Deshmukh et al., 2018	Small animal	Rat knee OA model (ACL transection + partial medial meniscectomy)	48	Small molecule (SM04690)	Inflammatory signaling pathway inhibition; Catabolic enzyme inhibition	- SM04690 is a Wnt pathway inhibitor - Rats were administered an intra-articular injection of SM04690 1 week post-surgery - SM04690 treatment was associated with increased cartilage thickness, enhanced Safranin O staining, and improved cartilage surface integrity compared to controls - There was also increased chondrocyte markers (SOX9, COL2A1, aggrecan, COMP), decreased catabolic markers (MMP-1, MMP-3, MMP-13, ADAMTS5) and decreased GAG breakdown and nitric oxide production	[84]
Pan et al., 2023	Small animal	Rat knee OA model (intra-articular injection of iodoacetic acid)	24	Stem cells	Cytokine modulation; Pro-apoptotic protein inhibition	- When umbilical cord-derived MSCs were co-cultured with chondrocytes, there was increased expression of IL-10, TGF-β1, ACAN, COL2A1, SOX9, and BCL-2, with decreased expression of BAX and BAD - Treatment with umbilical cord MSCs in a rat OA model resulted in reduced cartilage degradation and decreased levels of IL-1β, TNF-α, and MMP-13	[85]
L. He et al., 2020	Small animal	Rat knee OA model (intra-articular injection of sodium iodoacetate)	24	Stem cells	Cytokine modulation; Catabolic enzyme inhibition	- Exosomes were isolated from BMSCs - In vitro studies demonstrated that exosome administration resulted in decreased expression of IL-1β, with increased expression of COL2A1 and ACAN and decreased expression of MMP-13 and ADAMTS5 compared to controls - In vivo, intra-articular injection of BMSC-derived exosomes reduced OARSI scores, increased COL2A1 expression in local chondrocytes and the associated ECM, decreased expression of IL-1β, TNF-α and IL-6, and upregulated IL-10 expression compared to negative controls	[86]
Kondo et al., 2017	Large animal	Primate knee OA model (medial meniscectomy)	7	Stem cells	Cytokine modulation	- Synovial MSCs were isolated from all 7 subjects - A medial meniscectomy was performed on both knees, and four weeks later, synovial MSCs were transplanted onto one of the defects of each subject - At 16 weeks, the MSC-treated knees showed higher Makin’s histologic scores compared to untreated knees - MSC-treated knees demonstrated cartilage composition similar to intact cartilage	[87]
Jiang et al., 2014	Large animal	Primate knee OA model (Clostridium histolyticum injection)	27	Stem cells	Catabolic enzyme inhibition	- MSCs were isolated from subjects’ bone marrow and a portion was expanded to be used as polyclonal MSCs (P-MSCs), and the other portion was used in chondrogenic differentiation assays to produce chondrogenic clonal MSCs (sC-MSCs) - Subjects were injected with Clostridium histolyticum to induce knee OA - One week later, subjects received an injection of sC-MSCs, P-MSCs or normal saline - Subjects treated with sC-MSCs demonstrated higher Makin’s histologic scores and decreased expression of matrix metalloproteinases compared to P-MSCs and controls 24 weeks after treatment initiation	[88]
Copp et al., 2023	Clinical	Patients with knee OA	610	Stem cells	Cytokine modulation	- Systematic review encompassing 610 patient - MSC-treated patients experienced improvements in level of pain and functionality - MSCs were observed to have a regenerative and protective effect on cartilage	[89]
Chahal et al., 2019	Clinical	Patients with late-stage Kellgren–Lawrence knee osteoarthritis	12	Stem cells	Macrophage polarization; Cytokine modulation	- Non-randomized phase I/II clinical trial consisting of 12 patients receiving intra-articular injections of various dosages of BMSCs (1 × 10^6^, 10 × 10^6^, and 50 × 10^6^) - Patients demonstrated significant improvement in pain, stiffness and quality of life, particularly patients receiving high-dose BMSCs - There were no differences in MRI and T2 scores between the different dosages - There was a notable decrease in pro-inflammatory macrophages and IL-12 after injection, demonstrating the immunomodulatory effects of MSCs	[90]
L. X. Chen et al., 2008	Small animal	Rat knee OA Model (medial collateral ligament transection + partial medial meniscectomy)	90	Gene therapy	Cytokine modulation; Inflammatory signaling pathway inhibition; Catabolic enzyme inhibition	- NF-κB p65-specific siRNA delivered via an adenoviral vector reduced NF-κB activation, lowered IL-1β and TNF-α levels, and decreased MMP-13 expression - Significantly preserved cartilage proteoglycan content (Safranin-O), reduced chondrocyte apoptosis, and improved OARSI scores - Found that NF-κB suppression interrupts early OA inflammatory and catabolic pathways	[91]
De la Vega et al., 2025	Clinical	Human patients with radiographic knee osteoarthritis	9	Gene therapy	Cytokine modulation	- Single intra-articular AAV-IL-1Ra injection resulted in sustained IL-1Ra expression for 12 months with no serious adverse events - Found that minimal systemic vector spread and stable synovial transgene expression across all dosages - Patients noted improved pain and function, with no MRI evidence of structural deterioration - This study is the first demonstration that AAV gene therapy has the potential to be safe and durable in human OA joints	[92]
Capito & Spector, 2007	In vitro	Canine chondrocytes	–	Nonviral gene therapy	Cytokine modulation	- Type II collagen-GAG scaffold used as nonviral gene delivery vehicle - Plasmid IGF-1 incorporated via crosslinking to scaffold - Sustained local IGF-1 overexpression in chondrocytes - Increased GAG production and ↑ type II collagen synthesis - Increased chondrocyte-like morphology and matrix deposition compared to controls	[93]
C. Yang et al., 2019	Small animal	Rat knee OA model (full-thickness cartilage defect)	48	Gene therapy	Inflammatory signaling inhibition	- C-type natriuretic peptide (CNP) gene delivered to BMSCs via recombinant adenoviral vector - CNP-modified BMSCs loaded onto chitosan/silk fibroin (CS/SF) porous scaffolds - In vitro, researchers observed increased ACAN, COL2A1, and SOX9 expression and increased Alcian blue staining - In vivo, there was increased hyaline-like cartilage regeneration compared to controls with higher histologic scores, increased GAG deposition, and increased type II collagen staining	[94]

### 4.1. Bioceramics

Studies have shown that bioceramics are not merely passive osteoconductive substrates but also act as active immunomodulatory biomaterials that can influence macrophage phenotype, T-cell differentiation, and the broader immune microenvironment. Bioceramic scaffolds are not permanent constructs. Instead, they undergo controlled biodegradation in the aqueous environment of synovial fluid and, through surface hydrolysis, release biologically active ions such as copper (Cu^2+^), lithium (Li^+^), or cerium (Ce^3+^), depending on their composition. The goal is for the ceramic matrix to be ultimately resorbed and replaced by newly formed osteochondral tissue, which will contribute biologically and mechanically to the regenerating tissue. Zhai et al. conducted an in vitro study to assess the effects of lithium-doped calcium silicate (LCS) bioceramic scaffolds on mouse macrophages and rabbit chondrocytes. Ion release was observed to downregulate pro-inflammatory cytokines (TNF-α, IL-6, and IL-1β) and upregulate IL-10, ultimately inducing M2 macrophage polarization. The LCS bioceramic scaffold also enhanced chondrocyte proliferation and matrix production (type II collagen [COL2A1], aggrecan [ACAN]). This therapy promoted osteochondral repair, increased glycosaminoglycan (GAG) content, and improved interface organization [67]. Similarly, Lin et al. conducted a study in a rabbit OA model, implanting a copper-incorporated bioactive glass-ceramic (Cu-BGC) that sustained the release of Cu^2+^ ions. After implantation, macrophages shifted to an M2 anti-inflammatory phenotype, secreting IL-10 and enhancing chondrocyte matrix synthesis and mesenchymal stem cell (MSC) osteogenic differentiation. Cu-BGC significantly improved cartilage thickness, type II collagen deposition, and subchondral bone regeneration while reducing synovial inflammation [68]. 

Further research has provided a deeper mechanistic insight into how bioceramic degradation products actively shape immune responses within the arthritic joint. A central question has been how ion release from implanted scaffolds influences local immune cell behavior, particularly macrophage polarization. In a study by Zhao et al., 3D-printed scaffolds composed of bioactive glass (BAG) and β- tricalcium phosphate (TCP) were used to investigate how ionic dissolution products function as immunomodulatory signals. Scaffold degradation was found to produce a transient rise in extracellular calcium followed by a gradual decline. This dynamic calcium flux induced intracellular calcium oscillations in macrophages, which directly influenced polarization states [95]. 

Building on this concept of ion-mediated immune modulation, Deng et al. demonstrated that Ce-loaded bioceramics further address a key pathological driver of OA by targeting oxidative stress. Through Ce^3+^/Ce^4+^ redox cycling, these materials scavenge reactive oxygen species, thereby protecting chondrocytes from oxidative damage and matrix degradation characteristic of late-stage OA [69]. In parallel, ion-releasing silicate ceramics have been used to extend this immunomodulatory capacity to adaptive immunity, suppressing Th17 polarization, promoting Treg differentiation and attenuating autoimmune-mediated joint inflammation [96]. 

Collectively, the studies above demonstrate that Li-doped ceramics excel at chondrocyte maturation, Cu-BGC improves interface regeneration, BAG/TCP optimizes macrophage state transitions, and Ce-containing ceramics target oxidative-stress-mediated cartilage degeneration [69]. While therapeutically useful, bioceramics have limitations in targeting the osteoimmunologic environment in OA. Structurally, bioceramics are rigid constructs, and OA treatment requires penetration into a non-vascular environment with dense cartilage matrix. This combination can limit bioceramic particle distribution and make it difficult to achieve therapeutic concentrations [97]. Additionally, depending on the dissolution byproducts, these particles could worsen oxidative stress in the OA microenvironment and contribute to worsening joint inflammation. For example, when investigating the degradation patterns of polycaprolactone/β-tricalcium phosphate (PT), researchers found that the phagocytosis of PT degradation byproducts promoted M1 polarization and increased oxidative stress, ultimately negatively affecting tissue repair [98]. Because of these challenges, the development of bioceramic therapeutics requires careful construction [99]. 

### 4.2. Metal-Based Scaffolds

In OA, metal-based implants can be leveraged to improve cartilage regeneration by modulating the immune environment. Previously, integrating proteins and growth factors into scaffolds has been used to regulate the immune system, downregulate chondrocyte apoptosis, and enhance chondrogenesis. However, integrating peptides and growth factors is costly, and many molecules have short half-lives or become biologically inert under physiological conditions [70,100]. Thus, the stability and low cost of inorganic ions makes them an appealing alternative for OA treatment. 

In particular, metal ions have been incorporated into scaffolds to enhance cartilage regeneration and modulate the immune response. A nanofiber 3D scaffold containing epigallocatechin gallate and strontium ions demonstrated greater antioxidant activity compared to controls, enhanced cartilage matrix expression, reduced chondrocyte apoptosis, and suppressed pro-inflammatory cytokine expression, which could collectively facilitate cartilage regeneration [70]. Strontium, in particular, has been reported to enhance the M2 macrophage phenotype and inhibit pro-inflammatory cytokine production, suggesting its potential benefit in OA to mitigate the chronic inflammatory environment characteristic of this condition [101,102]. Additionally, manganese (Mn) and cobalt (Co) have been combined with akermanite microparticles (AKT-MPs) and have been shown to prevent the progression of OA. In vitro, Mn and Co-AKT-MPs decreased the expression of TNF-α, IL-1β, and IL-6, leading to downregulation of the M1 phenotype and demonstrating reactive oxygen species scavenging ability, with increased cellular catalase expression in the presence of hydrogen peroxide (H2O2) [73]. Furthermore, when injected into mice treated with intra-articular monosodium iodoacetate to induce knee OA, Mn and Co-AKT-MPs reduced reactive oxygen species in the joint and slowed cartilage degeneration, with increased expression of proteoglycans and type II collagen compared to untreated controls [73]. Additionally, this treatment influenced subchondral bone remodeling. While both Mn and Co-AKT-MPs improved subchondral bone volume fraction (BV/TV), only Co-AKT-MPs significantly increased trabecular separation and trabecular thickness and significantly decreased the bone surface-to-bone volume ratio on micro-CT [73].

Additionally, metal ions can be incorporated into scaffolds alongside bioactive molecules to enhance articular cartilage regeneration by recruiting MSCs. Liao et al. manufactured a decellularized cartilage extracellular matrix scaffold lysed and conjugated to Apt19s, which is known to recruit MSCs. The addition of magnesium (Mg) to the scaffolds supported enhanced chondrogenic differentiation of synovial MSCs and promoted the M2 macrophage phenotype [103]. Magnesium was also found to inhibit NLRP3 inflammasome complex activation, a signaling pathway involved in mounting an immune response to foreign material, which can lead to the release of pro-inflammatory molecules and chondrocyte pyroptosis [103,104]. The magnesium/Apt19s scaffolds led to increased proteoglycan and type II collagen deposition and upregulation of chondrogenic genes, including SRY-box transcription factor 9 (SOX9), ACAN, and COL2A1, compared to the scaffold alone and the Apt19s-conjugated scaffold [103]. 

The incorporation of metal ions in scaffolds can effectively modulate the chronic inflammatory environment in OA. However, despite their promise, metal ion-releasing scaffolds have several limitations for clinical translation. Ions released from scaffolds may be rapidly degraded by the local immune system, impeding their long-term therapeutic effects and necessitating specialized delivery systems for sustained release [105]. There has also been limited efficacy with single-ion scaffolds, such as magnesium, without optimizing delivery or combining them with other therapeutics [106]. Moreover, it has been observed that metal–organic frameworks (MOFs) and other metal-based scaffolds can be toxic at high concentrations, causing oxidative damage and cellular apoptosis, thereby limiting their therapeutic use [107]. Additionally, the sustained release of metal ions requires advanced scaffold designs, which in turn lead to a complex manufacturing process and limit scalability.

### 4.3. Hydrogels 

Hydrogels are a promising therapy for OA due to their utility as an effective delivery system for bioactive molecules and their ability to conform to the shape of the joint. For example, a polymer/gelatin hydrogel with enhanced tissue adhesion and embedded liposomes containing dimethyloxalylglycine, an immune regulatory agent, was shown to upregulate HIF-α pathways that contribute to chondrogenesis and to downregulate the pro-inflammatory environment by suppressing TNF pathways and M1 macrophage polarization [108]. As the OA environment is characterized by excess reactive oxygen species, which drives oxidative stress and potentiates a pro-inflammatory environment, accelerating cartilage degeneration, strategies to directly suppress reactive oxygen species are clinically valuable. Given this, Lu et al. developed a phenylboronic acid-crosslinked polyvinyl alcohol hydrogel that degraded when exposed to elevated reactive oxygen species to release sodium diclofenac, reducing oxidative damage and promoting the conversion to an M2 macrophage phenotype [75]. Additionally, after creating a full-thickness cartilage defect at the distal femoral condyle in a rat model, the authors observed increased reactive oxygen species clearance, upregulation of key cartilage repair genes (COL2A1 and ACAN), and notable cartilage regeneration in rats treated with the hydrogel [75]. 

Additionally, hydrogels can serve as delivery systems for stem cell-derived vesicles. For OA, hyaluronic acid-based hydrogels seeded with stem cell-derived exosomes have shown promise in reducing the pro-inflammatory environment and increasing the M2:M1 ratio to promote cartilage regeneration [74,75]. Sang et al. implanted thermosensitive hydrogels containing chondrocyte exosomes into a murine knee OA model and reported that their Pluronic F-127 and hyaluronic acid hydrogel could facilitate the delivery of exosomes into the joint cavity, reduce cartilage damage, and enhance matrix synthesis [74]. Further, Pang et al. developed a gelatin methacryloyl (GelMa) hydrogel loaded with MSC-derived nanovesicles (MSC-NVs). In vitro, the hydrogel promoted chondrocyte and bone marrow-derived stem cell (BMSC) proliferation, migration, and anabolic extracellular matrix synthesis, and strongly induced M2 macrophage polarization [109]. In a mouse model that underwent destabilization of the medial meniscus through transposition of the medial meniscotibial ligament, intra-articular treatment with this therapeutic significantly restored cartilage histology, reduced MMP and inflammatory cytokines expression, increased COL2A1 and ACAN expression, and shifted macrophages towards the M2 phenotype [109].

Hydrogels have also been shown to be effective in combination with gene therapy. Zhou et al. injected a dexamethasone sodium phosphate hydrogel containing a lentiviral (LV) vector encoding hyaluronan synthase 2 into a mouse OA model (anterior cruciate ligament transection), which resulted in decreased synovitis and delayed cartilage degeneration [110]. This specific hydrogel promoted endogenous hyaluronic acid expression, improving joint lubrication and reducing friction, and exerted immune regulatory effects by increasing M2 macrophage polarization and lowering pro-inflammatory cytokine levels (IL-6 and TNF-α) [110]. In combination, these features effectively suppressed synovial inflammation, suggesting the potential to promote cartilage repair and halt OA progression. 

Studies have also been conducted in large animal models. Xu et al. developed a chitosan (CTS) hydrogel loaded with icariin (ICA), a plant-derived compound known to have anti-inflammatory and chondrogenic properties. In a goat subcutaneous implantation model, the ICA/CTS hydrogel resulted in more native cartilage-like morphology and significantly increased GAG and COL2A1 expression relative to the CTS hydrogel [77]. In vitro, the ICA/CTS hydrogel enhanced chondrocyte proliferation and reduced the expression of inflammatory cytokines, including IL-6 and TNF-α, compared to CTS-only hydrogels [77]. Additionally, the use of hydrogels in horse models of OA may be relevant to human applications. Tnibar et al. injected horses with radiographically confirmed OA in either the carpal or metacarpophalangeal joint with a polyacrylamide hydrogel and observed a significant reduction in lameness scores and size of the associated joint effusion over the two-year study period [111]. Polyacrylamide is thought to have immune-modulatory properties due to its hydrophilicity [112]. Hydrophilic biomaterials have previously been reported to decrease pro-inflammatory cytokines, increase anti-inflammatory molecules, and positively influence cellular adhesion and proliferation, thereby promoting tissue regeneration [113,114,115,116]. 

Hydrogels, while promising for OA treatment, have notable limitations. Despite advances in design and manufacturing, hydrogel release kinetics can remain inconsistent, resulting in variable retention of seeded cells and delivery of therapeutic molecules [117,118,119]. Furthermore, most hydrogels lack the mechanical strength necessary to provide structural support, and their use in weight-bearing joints, such as the knee, can accelerate their degradation under mechanical loading before therapeutic efficacy is achieved [118,119]. Similarly, the highly inflammatory, reactive oxygen species-rich environment within an OA joint may further accelerate hydrogel breakdown [120]. This has led to the development of reactive oxygen species-sensitive hydrogels that, upon degradation, release anti-inflammatory molecules in response to elevated reactive oxygen species levels [121]. In some cases, the biocompatibility of hydrogels can also be a limitation. Compounds used in the polymerization of synthetic hydrogels can be toxic and may reduce cell viability [117]. This has contributed to the increased use of organic, plant-based compounds in the development of hydrogels [122]. Despite these challenges, hydrogels remain a viable delivery system for OA treatment due to their conformable structure and their ability to incorporate a variety of other therapeutic agents.

### 4.4. Nanoparticles and Microspheres

Nanoparticles (NPs) and microspheres have also emerged as innovative delivery systems capable of enhancing cartilage repair by targeting immune responses and releasing bioactive molecules [123,124,125]. Chronic inflammation in OA is sustained in part by a metabolic transition from oxidative phosphorylation to aerobic glycolysis in macrophages and chondrocytes, driven by cytokines such as TNF-α and IL-1β. This glycolytic transition worsens synovial inflammation, cartilage breakdown, and chondrocyte ferroptosis. Li et al. engineered a macrophage-membrane-coated nanoparticle that delivered IL-10 and nitric oxide (NO)-prednisolone to interrupt this glycolytic signaling cascade by modulating the IL-10, IL-10Rα, and the HIF-1α axis [78]. This treatment suppressed glycolysis, reduced reactive oxygen species and lactate accumulation, limited chondrocyte ferroptosis, and improved cartilage integrity in a murine knee OA model. Specifically, the study noted improved proteoglycan content (a protein within the ECM of cartilage which provides biomechanical support), improved Safranin-O staining (a marker of cartilage health), and lower cartilage degeneration scores [78,126].

Alternatively, microspheres that target two key drivers of OA pathology, acute cytokine inflammation and the persistent M1 macrophage phenotype, have been developed. Qiu et al. used a double-layer microsphere system in a rat knee OA model induced via medial meniscectomy, in which the outer layer of the therapeutic released a non-steroidal anti-inflammatory drug (parecoxib) to rapidly reduce inflammatory cytokine expression in the synovial fluid; the inner layer released macrophage-polarizing cytokine IL-4 over a prolonged period to drive macrophage conversion toward the M2 phenotype [79]. Compared to controls, the experimental groups demonstrated reduced levels of inflammatory cytokines (IL-1β and TNF-α), increased M2 macrophage markers in the synovium, and enhanced measures of cartilage matrix repair [79]. Additionally, Dai et al. developed a microfluidic hydrogel microsphere system and evaluated its utility in a rat knee OA model. The microspheres were loaded with folate-modified polymeric nanomicelles (FPTD) encapsulating sodium diclofenac. The folate ligand enabled preferential uptake by pro-inflammatory M1 macrophages, leading to M1 macrophage apoptosis and suppression of M1 polarization, while concurrently promoting the M2 phenotype. The authors noted reduced joint inflammation, preserved joint space width, improved cartilage histology with lower Mankin scores, and significantly increased cartilage matrix markers (type II collagen and aggrecan) [80]. Sulforaphane-loaded poly(lactic-co-glycolic acid) microspheres (SFN-PLGA) have also been constructed to provide immunomodulation in a rodent OA model. Ko et al. induced OA through ACL transection and administered an intra-articular injection of SFN-PLGA microspheres embedded in fibrin glue. The injection suppressed key osteoimmunologic and catabolic mediators, including cyclooxygenase- 2 (COX-2), ADAMTS-5, and MMP-2, effectively attenuating inflammatory and matrix-degrading pathways [81]. In vivo, the authors observed delayed cartilage degeneration compared to controls, as assessed by safranin-O staining, COL2A1 expression, and synovial hyperplasia and vascularity [81]. The study supported the use of SFN-PLGA microspheres as a potential disease-modifying treatment of OA. 

Despite their therapeutic utility, nanoparticles and microspheres also have several limitations. Given the continual flow of synovial fluid within the joint cavity, these particles are rapidly cleared, thus requiring frequent dosing to achieve therapeutic levels and increasing the risk of adverse effects [125]. Biocompatibility problems may also arise, as some metal nanoparticles have been found to aggravate inflammation or trigger oxidative stress. In addition, when introduced into the human body, nanoparticles are often identified as foreign entities, potentially creating a significant immune response after implantation [125]. To combat particle recognition, “stealth” coatings that employ polysaccharides or biomimetic membranes, be they platelet- or red blood cell-derived, have been developed to reduce immune recognition, limit cellular uptake, and improve the particle’s bioactivity [127]. For instance, Li et al. developed a macrophage membrane-coated IL-10/NO-prednisolone nanoparticle that incorporated this biomimetic “stealth” coating. The macrophage cloak reduced immune recognition, prolonged intra-articular residence, and preferentially allowed for binding to inflamed synovial macrophages. Zhou et al., similarly, developed M2 macrophage membrane-coated NPs able to create a robust M1 to M2 repolarization, suppress TNF-α and IL-1β, and reduce cartilage loss [128]. 

## 5. Molecular and Cellular Therapies

### 5.1. Small Molecules

An emerging area of research in the treatment of OA has focused on the administration of small molecules to target both its symptoms and the underlying disease mechanisms. Small molecules can directly modulate osteoarthritis pathways and the local immune microenvironment. Lohmander et al. developed an injectable therapeutic, Sprifermin, a form of recombinant human fibroblast growth factor 18 (rhFGF-18), which can induce chondrogenesis and cartilage matrix production and has also been found to increase type II collagen deposition and decreased MMP-13 expression [129]. In patients with knee OA, intra-articular injection of Sprifermin was associated with a reduction in lateral tibiofemoral joint space narrowing at 12 months compared with the placebo group [82]. However, this treatment did not lead to improvements in medial joint space narrowing or functional outcomes. Furthermore, in a meta-analysis of eight studies on the use of Sprifermin in knee OA patients, no adverse effects from these injections were reported, but it was also noted that Sprifermin was not associated with symptom relief [130]. Additionally, SM04690, a small-molecule Wnt pathway inhibitor, was found to be chondroprotective in animal models. Utilizing an ACL transection-induced OA model in rats, this study assessed the efficacy of this small molecule by administering a single intra-articular injection of 0.3 mg before surgery. Results showed increased cartilage thickness, upregulation of chondrogenic markers (COL2A1, aggrecan), and an increased number of articular chondrocytes. Catabolic mediators such as MMP-1, MMP-2, and ADAMTS5 were also reduced, suggesting that SM04690 promotes cartilage repair while inhibiting joint degeneration [84]. 

Recently, a small molecule has been manufactured to promote cartilage regeneration rather than just repair. Shkhyan et al. investigated the glycoprotein 130 (gp130) molecule and noted that inhibition of the non-canonical gp130 signaling mediated by the SRC family of kinases could influence cartilage regeneration. The IL-6 family is involved in the promotion of an inflammatory environment, and a key signaling protein within this pathway is gp130, with prolonged activation of this molecule thought to be involved in the pathogenesis of OA [83]. First, it was demonstrated that rats with genetically inactivated gp130 showed reduced proteoglycan loss, synovitis, and fibrosis compared with wild-type mice 6 weeks after surgical destabilization of the medial meniscus [83]. In addition, pharmacological inactivation of gp130 was induced through the intra-articular injection of R805, a small-molecule inhibitor acting on the gp130 signaling pathway. The administration of R805 in a rat knee OA model demonstrated improved OARSI scores and preserved cartilage thickness compared to negative controls [83]. In a canine knee OA model, similar results were observed with decreased synovitis, OARSI, pain, and lameness scores compared with controls [83]. In examining the underlying mechanism of R805, single-cell sequencing showed the promotion of anti-inflammatory macrophages and fibroblasts [83]. With these promising results, R805 (CX-011) is now being investigated in humans in a Phase 1/2A clinical trial.

One of the greatest challenges to the clinical application of small molecules is their safety profile. While these molecules have been observed to inhibit or stimulate specific pathways in cartilage degradation, repair and regeneration, they may also be involved in other pathways leading to off-target effects [83]. Further experimental trials are needed to better characterize this pharmacotherapy’s safety.

### 5.2. Stem Cell Therapy 

MSCs exhibit important immunomodulatory properties and can be derived from a variety of sources, including bone marrow, dental pulp, adipose tissue, and placental tissue. In response to highly inflammatory environments, MSCs have been shown to release pro-healing molecules, including prostaglandin E2, TGF-β, and IL-10 [131]. MSC-derived cytokines can also influence the development and proliferation of immune cells by promoting the transition from M1 macrophage polarization to the M2 phenotype [131]. Further, MSCs decrease the activation of dendritic cells, inhibit the proliferation of natural killer (NK) cells, and reduce antibody production by inhibiting B lymphocyte development [131]. Therefore, MSCs can help shift the inflammatory environment toward a regenerative state.

Various studies have been conducted to explore the molecular mechanism of stem cell-based therapies in the setting of OA. In chondrocytes co-cultured with MSCs derived from umbilical cord tissue, increased expression of key chondrogenic genes (COL2A1, SOX9, and B-cell lymphoma 2 [BCL-2]) and decreased expression of genes involved in cartilage breakdown (BCL2-associated X protein [BAX] and BCL2-associated agonist of cell death [BAD]) were observed [85]. MSCs also upregulated IL-10 and TGF-β1 expression, which suggests that MSCs have both regenerative and immunomodulatory effects. In rats treated with an intra-articular injection of iodoacetic acid, MSC therapy decreased levels of IL-1β, TNF-α, and MMP-13 and promoted cartilage regeneration by upregulating type II collagen and aggrecan [85].

The chondrogenic effect of MSCs has been tested in primate models. In an older cynomolgus primate model that underwent medial meniscectomy, the implantation of synovial MSCs was found to increase medial femoral condyle Mankin’s scores at 16 weeks post-implantation [87]. Furthermore, regenerated cartilage showed a similar composition of collagen, glycosaminoglycan, and water to that of intact cartilage compared to defects not treated with MSCs [87]. Similarly, Jiang et al. injected younger cynomolgus primates with collagenase to induce knee OA and treated subjects with an injection of chondrogenic clonal MSCs (sC-MSCs) or polyclonal MSCs (P-MSCs). Subjects treated with sC-MSCs demonstrated higher histologic scores and decreased expression of matrix metalloproteinases compared to P-MSCs and controls [88]. Overall, primate studies have demonstrated clinical potential of MSCs to treat osteoarthritis. 

Stem cells are already being used clinically in the treatment of OA, with a variety of human studies examining their immunomodulatory mechanism and their potential to improve patient outcomes. In a systematic review of 26 randomized and nonrandomized controlled trials regarding the use of MSCs in patients with knee OA, Copp et al. found that 12 out of 15 studies revealed improvements in patients’ functional outcomes and pain over the course of 6 to 24 months, while 17 out of 21 studies demonstrated evidence of cartilage regeneration or protection [89]. Similarly, Chahal et al. demonstrated that intra-articular injections of BMSCs improved patient-reported outcomes, including pain and quality of life in patients with knee OA without serious side effects over a 12-month period [90]. Notably, MSC administration led to decreased pro-inflammatory macrophages and IL-12. 

Nonetheless, stem cells still carry several risks including immunogenicity, tumorigenicity, and challenges with storage [86,132,133,134]. Moreover, many of the therapeutic benefits of stem cells are now thought to be due primarily to secretory factors rather than the stem cells themselves [86,132]. Because of these challenges with stem cells, there has been growing interest in the therapeutic use of stem cell-derived exosomes as an alternative therapy. Exosomes are extracellular vesicles that play a vital role in cellular communication [86]. Using BMSC-derived exosomes, He et al. demonstrated that the treatment of knee OA with exosomes could decrease the inflammatory environment and promote cartilage regeneration. In vitro, exosome administration decreased the IL-1β expression, an inflammatory cytokine known to impair chondrocyte proliferation and migration [86]. This was associated with the increased expression of cartilage anabolic markers, COL2A1 and ACAN, and decreased the expression of catabolic enzymes, MMP-13 and ADAMTS5, compared with IL-1β-treated controls [86]. In vivo, injection of BMSC-derived exosomes into the rat knee joint reduced OARSI scores on histology and decreased expression of IL-1β, TNF-α, and IL-6 with increased IL-10 expression compared with negative controls [86]. 

Despite their promise and current experimental use in OA treatment, stem cell-based therapies have several drawbacks. The chronic inflammatory environment characteristic of OA leads to decreased differentiation and proliferation capacity of endogenous stem cells [135]. Additionally, oxidative stress within the joint microenvironment decreases the proliferative capacity of injected MSCs, induces senescence and limits their overall immune modulation [136]. Beyond environmental limitations, stem cells themselves pose risks. Stem cells can be derived from autologous or allogeneic sources, each with its own advantages and risks. Autologous cells are harvested from a patient’s own cells, commonly from lipoaspirate or bone marrow, which requires an additional procedure and may cause iatrogenic pain and increase the risk of infection [137]. Conversely, allogeneic cells eliminate the need for a secondary procedure but carry the possibility of an immune reaction. If recognized as foreign, the immune system can mount a response, potentially increasing inflammation and exacerbating the degenerative process of OA [138]. As research on stem cells continues to advance, investigations aimed at mitigating these disadvantages are being explored. For example, the use of allogeneic stems in combination with immunosuppressive or immunomodulatory therapy is being used as a strategy to decrease immune reactivity [139,140]. Moreover, induced pluripotent stem cells (iPSCs) are being investigated to develop a universal donor to reduce immune rejection of cells as well as improve the availability of stem cell-based therapies [141,142]. Stem cells have significant clinical potential for the treatment of OA, but optimization of the cell source, cell dose and frequency of injection is necessary. 

### 5.3. Gene Therapy

Gene therapy strategies have taken two principal approaches in targeting OA through immune modulation: silencing or blocking catabolic, pro-inflammatory genes or overexpressing anabolic, anti-inflammatory mediators. An early study by Chen et al. demonstrated that knee OA progression could be suppressed by using an adenovirus (AV) vector to deliver siRNA targeting the NF-κB p65 subunit in a rat model. This intervention reduced NF-κB activation, lowered synovial fluid concentrations of IL-1β and TNF-α, attenuated synovial inflammation, and slowed cartilage degradation compared to controls [91]. More recently, a phase 1 clinical trial demonstrated that intra-articular expression of interleukin-1 receptor antagonist (IL-1Ra) was safe and sustained following adeno-associated virus (AAV)-mediated gene transfer in patients with knee OA [92]. The therapy achieved robust local IL-1Ra production for up to one year, improving baseline pain and functional outcome scores without significant systemic exposure or adverse events. Hydrogels have been used to deliver biologics that prevent cartilage degradation and promote repair. Alginate-based hydrogels containing viral vectors encoding insulin-growth factor-1 (IGF-1), a chondroreparative protein, have been found to enhance cartilage repair, increase COL2A1 expression, and prevent perifocal knee OA in a mini-pig model [143]. Capito et al. investigated the use of a type 2 collagen-GAG scaffold containing a DNA plasmid encoding IGF-1 to treat OA. IGF-1, from an osteoimmunology perspective, counteracts the inflammatory catabolism of OA joints [93]. In a study using cultures of adult canine articular chondrocytes, plasmid delivery resulted in sustained IGF-1 expression. This significantly enhanced chondrocyte proliferation, GAG accumulation, and type II collagen deposition, as shown by histology, which demonstrated increased tissue formation and chondrocyte-like morphology [93]. Furthermore, Yang et al. investigated AV-mediated C-type natriuretic peptide (CNP) gene-modified BMSCs seeded into chitosan/silk fibroin porous scaffolds to treat a rat full-thickness trochlear groove osteochondral defect model [144]. Compared with scaffold-only and unmodified BMSC controls, the modified constructs resulted in superior cartilage repair, with smooth articular surfaces, increased hyaline-like cartilage formation, and enhanced proteoglycan and type II collagen deposition [144]. These effects were attributed to CNP’s ability to antagonize IL-1β- and TNF-α-driven catabolic pathways, ultimately dampening NF-κB-mediated transcription of inflammatory mediators and matrix-degrading enzymes, downregulating RANKL expression, and limiting osteoclast activation and subchondral bone remodeling [144].

The limitations of gene therapy are notable. Immunogenicity is a significant barrier, as neutralizing antibodies to vectors can develop after initial administration, making re-dosing a significant challenge [145]. This is particularly relevant in OA, given its chronic and progressive nature. Age-related decline in transduction efficiency may also be a factor [146]. The target population for these therapeutics is older patients with reduced cellular uptake and expression of therapeutic transgenes, which may pose a barrier to gene transfer approaches [146]. Additionally, while a multitude of studies have shown successful use of vectors with sufficient safety profiles, transduction with vectors does pose a small risk of causing off-target effects, insertional mutagenesis, and inflammatory responses [145]. Finally, OA is a highly complex pathology, influenced by injury, obesity, and age. The variable phenotypes associated with OA should be considered when attempting to find a generalizable therapeutic [146].

## 6. Future Directions and Concluding Remarks

### 6.1. Translational Barriers

As osteoimmunology is an emerging field, research in this area remains limited. Many studies involving OA are conducted either in vitro or in small animal models, commonly mice and rats, with a relatively small number of large animal studies being performed. Further development of large animal models is needed to assist in the translation to clinical medicine. 

Moreover, animal studies may not translate clinically, due to important differences in human and animal immune system function and composition. For example, humans typically have 50–70% of neutrophils and 20–40% of lymphocytes in circulating blood, whereas rats, a common animal model used in OA and osteoimmunology research, have 10–20% of circulating neutrophils and 80–100% of lymphocytes [147]. Similarly, mice possess fewer IgG subclasses compared to humans and have differing macrophage biology, including the presence of the CD4 marker on human macrophages, which is absent in mice [147,148]. These interspecies differences in the immune system have led to the development of mice with humanized immune systems to better replicate human immune conditions in pre-clinical studies [149]. 

Other challenges with the clinical translation of animal studies are differences in disease progression, cartilage thickness, and biologic tissue repair. One issue is the differences in OA phenotypes with studies not distinguishing between post-traumatic OA, OA related to genetics, and age-associated OA [150]. Age is a key driver of osteoarthritic progression; however, many pre-clinical studies are conducted in younger animals and may therefore not fully represent the pathogenesis and disease progression in humans [150]. Additionally, rodents and other small-animal models have a thinner native cartilage layer with an increased number of chondrocytes when compared to human joints, which may increase their regenerative potential and healing capacity. [151,152]. Likewise, specific small-animal models, such as rabbits, have demonstrated the ability to spontaneously repair cartilage defects which may confound results of tested therapies [151]. Furthermore, gait patterns in many small- and large-animal models are not comparable to humans [151].The biomechanics and gait patterns of quadrupedal animal models further limit translatability. For example, canine and human knee joints differ in tendon and ligament anatomy, with canines having an additional lateral long digital extensor tendon that connects to the patellar groove, which may influence OA development and progression [151,152]. Further research is needed to develop more accurate animal models with OA characteristics and biomechanics comparable to humans.

Additionally, the implantation of any foreign material, including metal, hydrogels, or nanoparticles, can cause an immune response that has the potential to result in rejection or increased inflammation. Therefore, the development of therapies that have immune modulatory properties is critical, not only to regulate the highly inflammatory local environment of OA but also to prevent rejection and minimize inflammation in response to the implantation of these therapeutics. 

In general, across all therapies discussed in this review, there are large-scale manufacturing, long-term safety, and reproducibility challenges. Traditionally in the manufacturing of biomaterials, a “trial-and-error”-based approach is used; however, this is cost-ineffective and is a time-consuming process when increasing production and applying these therapies clinically [153]. Furthermore, differences among individuals such as immune status, metabolic differences, genetics, and variability in disease severity influence the effectiveness of individual therapies [153]. Moreover, there is the potential for foreign body reaction when any of these therapies are utilized. Different modifications have been developed to reduce immunogenicity such as bioactive coatings and PEGylation; however, new evidence demonstrates that these modifications may impact therapy efficacy and biodistribution [153]. Lastly, manufacturing capability is a significant challenge as many of the therapeutic solutions mentioned are structurally and compositionally complex, making it difficult to reproduce at a high quantity with Good Manufacturing Practice-compliant production [153]. There is also variability across batches with limited standardization that also influences clinical translation and regulatory approval [153]. In order to facilitate clinical translation, these issues in immunogenicity, scalability, and efficacy need to be addressed. 

### 6.2. Looking Ahead 

Many of these therapies can be used in combination, such as hydrogels that have the ability to deliver genetically modified stem cells [154]. Combining multiple therapies may improve efficacy as well as mitigate known limitations. For example, the use of hydrogels with other therapies can improve local delivery of genetically modified viral vectors, stem cells or anti-inflammatory molecules, diminishing the off-target effects of these therapies alone [154]. Yang et. al demonstrated that cartilage defects treated with genetically modified MSCs on a polymer scaffold resulted in improved cartilage repair compared to unmodified MSCs seeded on a polymer scaffold and that both of these treatments result in better healing compared to scaffolds alone [144]. Currently, gene therapy, small molecules and stem cells have been shown to be the most promising therapies when it comes to clinical translation and clinical trial progression (Table 2). A phase 1/2A clinical trial is underway for an anti-inflammatory small-molecule therapy, and stem cell therapy is presently being used clinically with mixed results. [89,155,156,157].

As the population ages and the prevalence of OA increases, innovative therapeutic strategies will likely integrate multiple approaches, such as scaffolds with genetically modified stem cells, to effectively enable cartilage regeneration and immune modulation. Future progress in osteoimmunomodulatory OA therapy will require a staged translational roadmap ultimately enabling patient-specific, clinically scalable, disease-modifying interventions.

## Figures and Tables

**Table 2:** Comparisons across therapeutic classes.

Therapeutic Strategy	Advantages	Disadvantages	Mechanisms of Action	Current Developmental Stage	Current Clinical Potential	References
Bioceramics	- Local delivery of therapeutic agents - Biodegradable	- Typically a rigid construct - Possibility of toxic breakdown products	Delivery of therapeutic molecules directly to the site of cartilage breakdown	Small animal	Low	[67,68,69,99]
Metal-based scaffolds	- Lower cost of metal ions compared to growth factors and biologic molecules - Provides mechanical stability	- Rapid degradation - Possibility of toxicity at high concentrations - Actual production of sustained metal ion release complicates manufacturing	Delivery of metal ions directly into the joint	Small animal	Low	[70,100,105,106,107]
Hydrogels	- Conforms to shape of joint - Can act as a delivery system for other therapeutic products (i.e., stem cells, viral vectors, etc.)	- Inconsistent release kinematics - Decreased mechanical strength - Complex manufacturing	Delivery of therapeutic molecules directly to the site of cartilage breakdown	Mainly small animal with some large animal studies	Moderate	[75,108,117,118]
Nanoparticles/Microspheres	- Can act as a delivery system for other therapeutic products (i.e., cytokines, metal ions, etc.) - Controlled elution of therapeutic molecules	- Rapid clearance of nanoparticles/microspheres - Decreased biocompatibility	Delivery of therapeutic molecules directly to the site of cartilage breakdown	Small animal	Low	[123,124,125]
Small molecules	- Act directly on OA pathways - Large range of therapeutic options	- Complex safety profile - Do not fully understand off-target effects	Act directly at joint space/cartilage interface to influence the inflammatory environment	Clinical	High	[82,83,130]
Stem cells	- Can directly provide chondrogenic cells for repair/regeneration - Can secrete a variety of naturally occurring biologically active molecules	- Autologous stem cells require harvesting - Allogeneic stem stems carry the risk of rejection/inflammatory response - Regenerative potential of stem cells can decrease with age and with increased inflammation	Cells can directly secrete anti-inflammatory molecules and can influence chondrogenesis	Clinical	High	[131,136,137,138]
Gene therapy	- Can target the underlying pathology - Ability to influence autologous cells which has the potential to have longer lasting effects - Large range of therapeutic options	- Concern for off-target effects of viral vectors - Possibility of developing antibodies against vectors - Complex manufacturing - Response to vectors can decrease with age	Influences gene expression by local cells	Wide range; mainly large and small animal studies with some clinical studies	High	[91,92,143,145,146]

## Data Availability

No new data were created or analyzed in this study. Data sharing is not applicable to this article.

## Appendix A

**Table A1:** Glossary of terms.

Term	Definition	Reference
*Compounds*		
Sodium diclofenac	A non-steroidal anti-inflammatory that can inhibit the NF- κB signaling pathway and increase the expression of cytokines such as IL-10 to increase M2 polarization	[75]
Apt19s	An aptamer, a short chain of nucleotides, that has an affinity for and can recruit MSCs	[103]
Pluronic F-127	A synthetic, biocompatible injectable hydrogel with thermosensitivity that positively influences cellular adhesion, collagen formation and angiogenesis	[158]
Icariin	A plant-derived compound from Epimedium plants known to have anti-inflammatory and chondrogenic properties	[77]
Dimethyloxalylglycine	A compound that induced hypoxic conditions, activating the HIF-1α pathway	[159]
Neobavaisoflavone	A plant-derived compound from Psoralea corylifolia that is used medicinally for its antioxidant, anti-inflammatory anti-cancer and anti-bacterial properties	[76]
*Cell Types*		
Th1	CD4+ T cell subtype; Helper T cell; Express IFN-γ	[160]
Th17	CD4+ T cell subtype; Helper T cell; Express IL-17	[160]
Th2	CD4+ T cell subtype; Helper T cell; Express IL-4, IL-5 and IL-13	[160]
Treg	CD4+ T cell subtype; Regulatory T cells; Promote tolerance and regulate the immune system to decrease autoimmunity and chronic inflammatory processes	[160,161]
Stem cell-derived exosomes	Cell communication vesicles that are paracrinally secreted by stem cells and contain compounds with regenerative potential including proteins, lipids, and genetic material	[162]
*Genes/Proteins*		
SRY-box transcription factor 9	SOX9: A key regulator within chondrocytes that plays a role in cartilage phenotype and maintenance of cartilage homeostasis	[163]
Aggrecan	ACAN: A proteoglycan that is a component of the cartilage matrix and helps to resists compression and disperse load	[164]
Type II collagen	COL2A1: The main component of cartilage that is responsible for the integrity of cartilage and is involved in chondrocyte proliferation and hypertrophy	[165]
Cyclooxygenase -2	COX-2: The rate-limiting enzyme in the production of prostaglandins and is upregulated during high levels of inflammation	[166]
Aggrecanase	ADAMTS-4, ADAMTS-5: Enzymes that are part of the metallopeptidase family and the breakdown of proteoglycan and aggrecan core proteins	[167]
Matrix metalloproteinase	MMP-1, MMP-3, MMP-13: The main enzymes involved in the breakdown of collagen and aggrecan	[167]
B-cell lymphoma 2	BCL-2: part of the Bcl-2 protein family; a protein that promotes cell survival through inhibition of apoptosis	[168]
BCL2-associated X protein	BAX: Part of the Bcl-2 protein family; a protein that promotes cell death through promotion of apoptosis	[169]
BCL2 associated agonist of cell death	BAD: Part of the Bcl-2 protein family; a protein that promotes cell death through promotion of apoptosis	[169]
Insulin-growth factor-1	IGF-1: A protein involved in the cartilage repair pathway; involved in cellular proliferation, differentiation and survival	[143,170]
T-cell factor/lymphoid enhancer factor	TCF/LEF: Downstream gene transcription factors in the Wnt/β-catenin signaling pathway	[171]
*Scoring systems*		
Mankin system	A histological grading system to evaluate articular cartilage degeneration based on structural integrity, cellularity, matrix staining (proteoglycan loss), and tidemark integrity	[172]
OARSI system	A histological grading system for cartilage degeneration based on lesion size and depth	[173]
Kellgren–Lawrence system	A radiologic grading system to classify the severity of OA based on the presence of joint space narrowing, osteophytes, subchondral cysts, and subchondral sclerosis	[174]
Western Ontario and McMaster Universities Osteoarthritis Index	WOMAC: A self- administered questionnaire to measure functional outcomes in patients with osteoarthritis evaluating pain, stiffness and impact on activities of daily life	[175]
Visual Analog Scale	VAS: A questionnaire to evaluate patient stated pain levels	[176]
Lequesne Functional Index	LFI: An interview administered questionnaire to measure level of pain and functional disability in patients with osteoarthritis	[177]
*Molecules*		
Interleukin-1	IL-1: Pro-inflammatory cytokine primarily produced by macrophages that initiates acute inflammatory responses and boosts lymphocyte activity	[178]
Interleukin-4	IL-4: Anti-inflammatory cytokine, primarily produced by Th2 cells that dampens inflammation by inhibiting pro-inflammatory cytokines and polarizing macrophages toward an anti-inflammatory M2 phenotype	[179]
Interleukin-6	IL-6: Promotes specific differentiation of naïve CD4+ T cells, linking innate to acquired immune responses; in combination with TGF-β, it is involved in Th17 differentiation from naïve CD4+ T cells; inhibits TGF-β-induced Treg	[180]
Interleukin-10, 10ra	IL-10: Potent anti-inflammatory cytokine that plays a crucial role in preventing inflammatory and autoimmune pathologies	[181]
Interleukin-12	IL-12: Critical in antigen presentation and influencing cell-fate decisions of differentiating naïve T cells	[182]
Interleukin-13	IL-13: Anti-inflammatory cytokine that antagonizes Th1-driven pro-inflammatory immune response; downregulates synthesis of many pro-inflammatory cytokines and chemokines	[183]
Interleukin-17	IL-17: Potent pro-inflammatory cytokine family produced primarily by Th17 cells, acting as a bridge between innate and adaptive immunity	[184]
Damage-associated molecular patterns	DAMPs: Endogenous danger signal molecules released by damaged, stressed or dead cells that activate immune responses and inflammatory signaling pathways	[185]
Tumor necrosis factor-α	TNF-α: Inflammatory cytokine produced by macrophages/monocytes during acute inflammation	[186]
Interferon-γ	IFN-γ: Pro-inflammatory cytokine crucial for innate and adaptive immunity, produced by Th1 cells and NK cells; activates macrophages and modulates immune responses	[187]
Granulocyte-macrophage colony-stimulating factor	GM-CSF: Important hematopoietic growth factor and immune modulator that stimulates the bone marrow to produce granulocytes and macrophages	[188]
Prostaglandins	PGE2, PGD2, etc.: Lipid autacoids derived from arachidonic acid; sustain homeostatic functions and mediate pathogenic mechanisms, including inflammatory responses	[189]
Transforming growth factor -β	TGF-β: A cytokine that is involved in chondrocyte differentiation and has anti-inflammatory or pro-inflammatory properties depending on environmental factors such as age of the patient and joint loading	[190]
Fibroblast growth factor-18	FGF-18: A cytokine involved in chondrogenesis and cartilage repair; has been found to influence cartilage degeneration through increasing type II collagen deposition, reducing MMP-12 expression and decreasing IL-1β-induced apoptosis	[129]
*Pathways*		
Wnt/β-catenin	Wnt/β-catenin; signaling by the Wnt family of secreted glycolipoproteins is one of the fundamental mechanisms that direct cell proliferation, cell polarity and cell fate determination	[51]
Mammalian target of rapamycin	mTORC: Serine/threonine kinase that controls cellular metabolism, catabolism, immune responses, autophagy, cell survival, cellular proliferation, and cellular migration to maintain cellular homeostasis	[191]
NOD-, LRR- and pyrin domain-containing protein 3 inflammasome complex activation	NLRP3: Component of the innate immune system that mediates caspase-1 activation and the secretion of pro-inflammatory cytokines IL-1β/IL-18 in response to infection or cellular damage	[192]
